# Moral distress: sometimes, there is no fix

**DOI:** 10.1007/s11019-026-10343-5

**Published:** 2026-03-19

**Authors:** Cullin Brown, Jan Helge Solbakk

**Affiliations:** 1 Centre for Medical Ethics, University of Oslo, Oslo, Norway; 2 Department of Philosophy, Binghamton University, Binghamton, NY USA

**Keywords:** Moral distress, Moral constraint, Moral experience, Healthcare providers

## Abstract

Since Andrew Jameton introduced the term “moral distress” (MD) in 1984, the idea has gained enormous attention in the healthcare literature. We offer a critical, narrative review of the understandings of MD that have been proposed, with an eye towards their scope and corresponding ability to speak to the moral vulnerability inherent in human life. Jameton’s understanding of MD is narrow in the sense that it requires the presence of institutional constraints (e.g., hierarchies or hospital policies) that prevent a healthcare professional from doing what they take to be morally right. Other theorists have suggested that internal constraints (e.g., lack of moral courage) can engender MD as well, and still others have suggested that neither kind of constraint is necessary, as MD can arise from moral conflicts and moral uncertainty in addition to constraints. We criticize understandings of MD that emphasize institutional and/or internal constraints, as they exclude cases in which MD does not arise from any flaw or shortcoming and so for which there is no fix. However, we also criticize understandings of MD that look beyond constraints in general, as they lose sight of MD’s distinguishing feature as a moral experience, namely the anguished or panicked sense that one is constrained from doing what one takes to be morally required. We suggest that the corrective involves acknowledging a third kind of constraint: *human constraints*, or constraints that arise from the human condition rather than any kind of flaw or shortcoming.

## Introduction

In 1984 Andrew Jameton introduced the term “moral distress” (MD) to capture a specific kind of distress that healthcare professionals (HCPs), in particular nurses, are prone to experience: “*Moral distress* arises when one knows the right thing to do, but institutional constraints make it nearly impossible to pursue the right course of action” (p. 6).^1^ Since then, MD has gained enormous attention in the healthcare literature. We offer an overview and critique of how MD has been defined since Jameton introduced the term, with an eye towards their scope and corresponding ability to speak to our moral vulnerability as human beings.

To preview our focus and position: There is widespread agreement that MD involves negative emotional responses such as anguish and panic (if it didn’t, it wouldn’t be a kind of *distress*) and that these responses must arise in a morally charged context (if they didn’t, they wouldn’t amount to *moral* distress). There is substantial disagreement, however, about how to characterize these two pieces and how they fit together. Much of this disagreement–and the part that we will focus on–pertains to the role and status of *constraints*. Jameton takes constraints, and specifically institutional constraints such as hierarchies and hospital policies, to be a necessary condition: MD only arises when such constraints prevent an HCP from pursuing the right course of action. Other theorists agree with Jameton that constraints are necessary but hold that internal constraints, or constraints that arise from within the HCP rather than the institutional context in which they work (e.g., lack of moral courage), can engender MD as well. Still others deny that constraints are necessary on the grounds that MD can arise in situations that are characterized by moral uncertainty and moral dilemmas rather than either type of constraint.

We think theorists who emphasize constraints have their finger on an important moral experience and that this experience speaks to our moral vulnerability as human beings–our fragile and precarious condition as beings whose “capacity for care often exceeds [our] capacity for action” (Batavia et al. 2020, p. 1119). However, we also think these theorists characterize constraints in a way that excludes certain instances of this experience and so fail to capture the depth of our moral vulnerability. Alternatively, theorists who emphasize moral dilemmas and moral uncertainty in addition to constraints catch some of the relevant cases but lose sight of what unifies them as instances of the same moral experience–namely, we suggest, the felt collision of a moral “*I must*” with an “*I can’t*.” Accordingly, we characterize MD as *the panicked or anguished sense that one is constrained from doing what one takes to be morally required*, a characterization that both captures the relevant instances and keeps a finger on MD’s distinctiveness as a moral experience. We also suggest that there is a kind of “*I can’t*” that arises from the human condition rather than any flaw or shortcoming, which we call *human constraints*.

A methodological note: This paper was initially conceived as a systematic review, aimed at categorizing the many definitions of MD that have been proposed since Jameton’s 1984 account.^2^ Accordingly, it began with a search of Scopus (a multidisciplinary database that covers nearly all of PubMed/Medline content) for “moral distress” and related terms, combined with terms for HCPs and their practices.^3^ As we sifted through the publications identified by this search, however, we became less interested in any kind of bottom-up taxonomy of understandings of MD and more interested in how these understandings, by virtue of their differing scopes, shed light on our moral vulnerability as human beings. Because situating definitions of MD against this backdrop is more of a narrative than systematic endeavor, we changed course and adopted a looser methodology: we screened the abstracts of publications identified by our initial and subsequent searches for indications that they might advance a new or refined understanding of MD, read the papers with promising abstracts, and then retained those that we judged to in fact advance a new or refined understanding of MD.^4^ Finally, we grouped these understandings based on the extent to which they shed light on the moral vulnerability inherent in human life by helping articulate a particular kind of moral experience. Thus, we maintain that our survey of understandings of MD is representative–and we have left certain systematic remnants in order to support this assertion–but we do not claim that it is exhaustive, and we acknowledge that our groupings are more associative than analytic.

## What kind of thing is MD?

The first group of views that we will consider takes institutional constraints to be an essential feature of MD. However, surveying these views quickly raises a question that merits independent treatment, namely the question of what *kind* of thing MD is. Thus, in this preliminary section we will introduce these views only insofar as is necessary to raise and address this question.

The views in this group preserve the core elements of Jameton’s view–according to which MD “arises when one knows the right thing to do, but institutional constraints make it nearly impossible to pursue the right course of action” (1984, p. 6)–and generally aim to flesh out one or more of these elements. Wilkinson (1989), for example, elaborates on the psychological dimensions of MD, specifically the “negative feelings and psychological discomfort/disequilibrium” that HCPs tend to experience when institutional constraints prevent them from doing what they take to be right, as well as the coping mechanisms that HCPs use in an effort to “restore psychological equilibrium and wholeness” (p. 516).

Wilkinson’s focus on the psychological dimensions of MD raises a conceptual question that, as far as we can see, remains unsettled (and to our frustration, is seldom explicitly addressed). The question is what *kind* of thing theorists are talking about when they’re talking about MD. Answering this question is essential, as an assessment of the competing accounts of MD can only gain footing once it is clarified what a satisfactory account of MD should explain. In other words–and as other theorists have emphasized–“moral distress” is a term of art, and theorists are ultimately free to define terms however they wish; thus, an understanding of MD “cannot be motivated by an appeal to the meaning of ‘moral distress’” as a piece of natural language (Campbell et al. 2016, p. 3). Rather, such an understanding must be motivated (or critiqued) on other grounds. For example, Wilkinson’s emphasis on psychological disequilibrium and coping mechanisms that might restore wholeness suggests that she is thinking about MD as a *psychological state* (as we will explain shortly, though, this is not the full story with Wilkinson), and that a good understanding of MD should therefore characterize this state in terms of its eliciting conditions, physiology, valence, function, regulation, and the like.^5^ Approaching MD as a psychological state therefore supplies one backdrop against which an understanding of MD can be assessed; it anchors claims such as that a (hypothetical) understanding of MD is unduly narrow because circumstances other than institutional constraints frequently elicit psychological states with the same physiology and function.

Yet insofar as Wilkinson understands MD as a psychological state, she already departs from Jameton in an important way. Jameton emphasizes institutional constraints at least in part to characterize a *moral problem*, namely that healthcare institutions systematically constrain HCPs’ moral agency (1984, pp. 7–15). As theorists have subsequently emphasized, moreover, this moral problem is tied to a host of negative consequences: it can erode HCPs’ wellbeing, undermine quality of care, and contribute to staff turnover and shortages (Corley 2002; Gutierrez 2005; Prompahakul et al. 2006; Hamric and Blackhall 2007; Goethals et al. 2010; Whitehead et al. 2015; Epstein 2020; Carletto et al. 2022). Because Jameton’s emphasis on institutional constraints is tied to his call for institutional reform, though, it is not quite clear to what extent he and those writing in his wake think institutional constraints are essential to the characterization of any particular psychological state, as opposed to the characterization of a problem that (incidentally) involves certain psychological states. Thus, it is not quite clear, either, to what extent the critique that circumstances other than institutional constraints can engender similar psychological states talks past these theorists.

We suspect that MD has captured the attention of so many at least in part because it points to something beyond a problem that needs to be addressed. Speaking simply as human beings, there is something deeply resonant about the experience, say, of a nurse who takes the continued aggressive treatment of a terminally ill patient to be wrong on the grounds that it is causing the patient unnecessary suffering, but who lacks the authority to resist a physician’s order to continue treatment. Moreover, what resonates here does not seem to be exhausted by our sensitivity to the need for institutional reform, but rather centers on our ability to grasp the nurse’s *experience*; we would nod along in pained recognition if the nurse described their experience as the moral analogue of being underwater and so unable to take the breath that one needs. Thus, although we readily acknowledge the moral and practical need for institutional reform, we think these needs are downstream from a more fundamental matter that the literature on MD has helped articulate.

We will therefore proceed under the assumption that institutional constraints should only figure in the definition of MD if they are integral to the characterization of this more fundamental matter. Once one adopts this assumption, though, there is still the question of what kind of more fundamental thing MD is. One possibility, as we have already noted, is that MD is a psychological state. This has arguably become the dominant view in the literature. Although rarely explicitly expressed, it is in background of the many efforts to quantify various features of MD (see, for example, Corley 2002; Corley et al. 2005; Epstein and Hamric, 2007; Hamric et al. 2012; and Rushton et al. 2013). To be sure, many of these efforts still emphasize institutional constraints, but they tend to do so because of the (perhaps unique) role that such constraints play in eliciting the relevant psychological states.

We do not object to this psychological emphasis in principle, but we think it has been pursued in an impoverished way. While scholars have explored MD’s elicitors, physiology, affect, regulation, etc., they have said comparatively little about its phenomenology–to borrow Nagel’s (1979) phrase, “what it is like *for* the experiencing organism” (p. 172). They have been especially quiet about its *moral* phenomenology, or its structure as a moral experience. Wilkinson (1989) cautions against this omission and the reductive psychological approach that underwrites it when she asserts that “moral distress has situational, cognitive, and action dimensions as well as the feelings dimension” (p. 24). Yet her assertion has received little uptake: MD is routinely treated as a negatively valenced mental state caused by moral circumstances rather than a distinctively moral lived experience.

When we nod along with the (imagined) nurse’s description of MD as the moral analogue of being unable to take the breath that one needs, we are recalling times when we have felt similarly and/or imaginatively stepping into the nurse’s standpoint and experiencing an approximation of what they are experiencing. The example therefore resonates–at least for us–because of our familiarity with a particular moral experience. This experience, moreover, cannot be captured in the third-personal terms of elicitors and physiology but requires first-personal statements–specifically, we will suggest, a moral “*I must”* and an “*I can’t.*” Accordingly, we will assume that the more fundamental matter that the literature on MD has helped articulate is a *distinct moral experience.* This experience is distinct insofar as its instances share a common experiential core that is sufficiently determinate to enable those who have had the experience to communicate about it without talking past one another.^6^ Moreover, this experience is *moral* in that an adequate account of its phenomenology must make recourse to moral concepts; put differently, someone without such concepts would not be able to fully grasp the experience.^7^

Our suggestion that MD is best understood as a distinct moral experience is not in tension with the psychological approach, but it does aim to enrich it by balancing the third-personal claims that comprise extant accounts with first-personal claims, and specifically first-personal moral claims. This may appear to be an unfair characterization or an unneeded correction, as researchers who study MD sometimes rely on the self-reported emotions of HCPs. However, when these self-reports are reduced to fodder for third-personal characterizations of MD (e.g., claims about which elicitors tend to provoke the most intense feelings)–and when scales are developed with an eye towards supporting this type of claim–such self-reports are only first-personal in a superficial sense. Moreover, we take the first-personal claims that comprise the moral phenomenology of MD to have an important claim to priority, as they are what make more objective investigations of MD meaningful in the first place. Thus, we maintain that the first task of an adequate understanding of MD is to articulate it as a moral experience and thereby shed light on our condition as beings who are susceptible to having such experiences.

This framing anchors claims such as that a particular understanding of MD is unduly narrow because it excludes certain instances of the relevant moral experience, an exclusion that is especially problematic insofar as it lends itself to a mischaracterization of our condition as human beings. Indeed, this is precisely the claim that we will soon make about the group of views that takes institutional constraints to be an essential feature of MD. First, though, we wish to acknowledge that this framing requires us to work with a modified version of Jameton’s view. Jameton characterizes MD as arising “when one *knows* the right thing to do” (1984, p. 6; emphasis added), and this characterization is consistent with his understanding of MD as a moral problem. That is, because institutional constraints are only morally objectionable when they prevent one from doing what *is* right, and because knowing the right thing to do involves having *true* moral beliefs or an otherwise *correct* moral take,^8^ Jameton is entitled to the strong language of “knowing.” However, such language is inconsistent with our understanding of MD as a moral experience, since false beliefs feel the same as true beliefs (and incorrect takes feel the same as correct takes), as long as they’re held with similar levels of confidence and sincerely one’s own. Our framing of MD as a moral experience therefore requires us to recast Jameton’s knowledge clause in terms of what an HCP *takes* to be right. Importantly, this is not to say that the correctness of HCPs’ moral conclusions is irrelevant, but that its relevance is downstream from the characterization of MD itself. By way of analogy: whether or not the target of one’s fear actually poses a threat to one is certainly important, but it does not bear on whether one is genuinely afraid.

## Institutional constraints

One family of views follows Jameton in holding that institutional constraints are a necessary condition of MD (see Table 1). Jameton (1984) identifies a range of such constraints, including hierarchical authority structures (and specifically physician dominance over nurses), hospital policies (e.g., policies requiring the continuation of treatment, even when such treatment is arguably wrong or harmful), and resource constraints (e.g., staffing shortages and time pressures that prevent HCPs from providing adequate care). Theorists later emphasized additional institutional constraints, such as cost-containment (i.e., resource constraints that are driven by economic considerations) and legal liability pressures (see, for example, Wilkinson 1987 & 1989; Corley, 1995; 2002; Rushton 2006; and Epstein and Hamric 2009). These are important refinements. Our focus, however, will be on the range of cases that these views exclude by treating institutional constraints as necessary, and the corresponding way in which these views entrench an understanding of MD as arising exclusively from problems that might, in principle, be remedied.

**Table 1:** Papers retained for analysis

Category	Number of papers	References
External/institutional constraints	25	Jameton (1984); Wilkinson (1989); Jameton (1993); Tiedje (2000); Corley (2002); Lützen et al. (2003); Hardingham (2004); Corley et al. (2005); Rushton (2006); Hamric and Blackhall (2007); Schluter et al. (2008); Austin et al. (2008); Iglesias et al. (2012); Ganz et al. (2013); Sunderland et al. (2015); Lachman (2016); Sasso et al. (2016); Schaefer et al. (2016); Fantus et al. (2017); Prestia et al. (2017); Fischer-Grönlund and Brännström (2021); Molazem et al. (2022); Rosa et al. (2022); Girela-Lopez et al. (2024); Belfi et al. (2024).
Internal/agential constraints	19	Wilkinson (1989); Nathaniel (2002); Epstein and Hamric (2007); Pendry (2007); Lützen and Kvist (2012); Carnevale (2013); Rushton et al. (2013); Thomas and McCullough (2014); Zavotsky and Chan (2016); Fumis et al. (2017); Sara Rosenthal and Clay (2017); Berger et al. (2019); Porr et al. (2019); Welborn (2019); Berhie et al. (2020); Binkley and Kemp (2020); Marturano et al. (2020); Woods (2020); Beyaffers (2021)
Broader accounts	14	Hanna (2004); McCarthy and Deady (2008); Lopez (2010); Bennett et al. (2013); Fourie (2015, 2017); Campbell et al. (2016); Monteverde (2019); Kada and Lesnik (2019); Morley et al. (2019; 2020); Ujewe (2020); O’Hanlon et al. (2021); Tarabeih and Bokek-Cohen (2021)
Total	58	

Given this focus, Jameton’s view is significant both conceptually and historically. Jameton characterizes MD in part by differentiating it from moral dilemmas and moral uncertainty, which he defines as follows: “*Moral uncertainty* arises when one is unsure what moral principles or values apply, or even what the moral problem is… *Moral dilemmas* arise when two (or more) clear moral principles apply, but they support mutually inconsistent courses of action” (1984, p. 6). This contrast matters because although some instances of moral uncertainty and moral dilemmas arise from problems that might be remedied–for example, uncertainty rooted in an HCP’s moral incompetence or a dilemma that better resource allocation could have prevented–few would maintain that *all* such cases are avoidable, even in principle. Imagine, for example, an HCP who is uncertain about the moral status of aggressively treating a severely impaired newborn with poor prognosis. Such an HCP might be uncertain about what counts as a benefit or harm to the newborn–or even about whether these notions can be meaningfully applied in this kind of situation–as well as about how to navigate the sometimes fine line between fair resource allocation and discrimination against disability. We take the HCP’s moral uncertainty in this kind of case to reflect the indeterminacy that is inherent in our moral principles rather than any kind of flaw or shortcoming, a point underscored by the fact that we would look poorly upon, or at least have serious reservations about, an HCP who approached such a case with too much confidence.

Imagine, too, a case in which a young child would benefit from treatment for a life-threatening disease, but the child’s parents object to the treatment on religious grounds (for instance, blood transfusions for Jehovah’s Witnesses). The requirement to respect the right of parents to make healthcare decisions for their underage child conflicts with the obligation to save the child’s life, and the HCP therefore faces the dilemma of choosing which moral requirement to uphold and which to violate. And here again, there is arguably no way to prevent this kind of case from arising, at least while respecting deep differences in human values (i.e., even if one dismisses the parents’ values as unreasonable in this particular case).

These examples serve an underappreciated existential function in addition to their more familiar function of fostering ethical reflection: they help situate moral uncertainty and moral dilemmas as unavoidable features of human life–features that we must, at some level, accept rather than resolve. By characterizing MD in terms of institutional shortcomings and in opposition to moral uncertainty and moral dilemmas, then, Jameton tacitly portrays it as something *fixable*, and therefore as something peripheral to the human condition. We think this is a misstep, as the experience of the HCP in either of the examples just described may share an experiential core with that of an HCP who is prevented, by institutional constraints, from pursuing the course of action they take to be right. Attending to this experiential core, moreover, suggests that not all instances of MD are preventable and that our susceptibility to MD therefore speaks to the depth of our moral vulnerability as human beings.

Consider, first, that the HCP in all three situations is likely to have what Street (2008) calls a “normative experience,” or an experience of something in the world–for instance, a suffering patient–“as ‘counting in favor of’ or ‘calling for’ or ‘demanding’ certain responses on [their] part” (p. 240). More specifically, the HCP in all three situations is likely to have at least one *moral deontic experience*–that is, an experience of a certain response as either morally required or morally prohibited. This contrasts with non-moral deontic experiences (such as experiences of professional requirements), as well as moral and non-moral evaluative experiences, in which a response is experienced as good or bad but not required or forbidden (e.g., morally admirable but not obligatory). In the example of moral uncertainty, the HCP may feel morally forbidden from wronging or failing the newborn, but unsure what exactly would amount to wronging or failing them and so prohibited from pursuing any course of action. Conversely, the HCP may feel morally required to do *something* for the newborn–it is clear to them that the struggling newborn demands some kind of active response–but utterly unsure what that something is. Similarly, the HCP in the moral dilemma example may experience requirements both to treat the child’s life-threatening condition and to respect the parents’ wishes. Finally–and continuing with the example of the nurse who takes continued aggressive treatment of a patient to be wrong but who lacks the authority to stop or scale back treatment–the nurse may experience a moral requirement to ensure that the patient lives the remainder of their life with minimal suffering and/or a prohibition against causing unnecessary suffering.

Second, in all three situations the HCP is constrained from acting in at least one of the ways they experience as required (or from refraining from doing what they experience as forbidden).^9^ In the example of moral uncertainty, it would be out of the question for the HCP to remain idle; thus, they must commit to a course of action despite feeling prohibited from doing so, given that every course of action risks failing or wronging the newborn. Time pressures and the duty to act therefore prevent the HCP from heeding their experience of moral prohibition. Similarly, the HCP caught in a moral dilemma is prevented from fulfilling either obligation by the competing obligation; as soon as the HCP turns towards one, the other presents itself as a constraint. Moreover, even if the HCP experiences one requirement as overriding, they may still experience the other as binding, and so as a constraint against acting on the first requirement (in much the same way, to borrow Williams’ [1973] example, that the desire to spend “Christmas in Christmassy surroundings in Austria” can exist alongside the overriding desire–and consequent decision–to spend “it back home in the familiar Christmas heat of [one’s] birthplace” [p. 167]). Finally, the nurse in the Jameton-style case is of course constrained by institutional hierarchies that deprive them of the authority to resist the physician’s order to continue aggressive treatment.

The experiences of the HCPs in these three situations therefore share a common structure: each involves a moral deontic experience paired with an experience of constraint–more colloquially, a moral “*I must*” alongside an “*I can’t*.” The question, then, is whether this structure captures the core of the nurse’s experience in the Jameton-style case. If it does, it captures the experiential core of MD (because those who emphasize institutional constraints acknowledge this kind of case as paradigmatic). But in that event, one must acknowledge that MD can also arise in response to moral uncertainty and moral dilemmas, since the same structure can characterize one’s experience in those cases as well.

We think this structure does capture the core of the nurse’s experience in the Jameton-style case, and therefore that MD can arise in response to moral uncertainty and moral dilemmas as well. This is not the sort of claim that can be rigorously established, since whether a structure captures the core of an experience can only be settled from the first-person perspective, where what counts as central rather than incidental depends in part on one’s values and associative history. Still, we find it intuitively compelling–true to our experience–that this structure is the defining feature of the many examples and case studies of MD in circulation.

The most pressing objection that we foresee to this claim is that the nurse in the Jameton-style case is likely to experience blame or resentment directed at some other person or entity–in this case, the physician who orders continued aggressive treatment–that the HCPs in the other examples likely will not (Rushton 2006, p. 162; Rushton et al. 2013), and that such interpersonal responsibility responses are partially constitutive of MD. We suspect, however, that this is an additional experiential layer rather than part of the core structure of MD. Imagine, for instance, that the HCPs from the three examples are sharing their experiences with each other and that the nurse from the Jameton-style case tells the others, “At least I have someone to blame; you don’t even have that.” Such a remark presupposes a shared experiential core and suggests that interpersonal responsibility responses are incidental to MD, perhaps even something that can lessen one’s distress by giving it an outlet. What varies across the three cases, then, is not their experiential core but the layering (or lack thereof) of other experiences on top of it.

We therefore think Jameton was mistaken to distance MD from moral uncertainty and moral dilemmas, as these phenomena can engender MD, and thus all three must, at some level, be accepted rather than remedied. Yet because Jameton also drew attention to an underappreciated problem and helped articulate an important and undertheorized moral experience, his characterization of MD was quickly and widely embraced. Thus, the many refinements and elaborations that soon followed–including some by Jameton himself–do not invite fresh objections so much as further entrench his original missteps.

For example, in response to Wilkinson (1987), Jameton distinguishes between initial and reactive moral distress:

Initial distress involves the feelings of frustration, anger, and anxiety people experience when faced with institutional obstacles and conflict with others about values. Reactive distress is the distress that people feel when they do not act upon their initial distress. Wilkinson… emphasized the reactive distress of possible inaction, and I identified the initial distress of facing conflict with others over moral issues. (Jameton 1993, p. 543)

This distinction builds out the taxonomy of MD, but it leaves intact the assumption that MD arises specifically from institutional constraints. Similarly, Jameton suggests that situations of MD can give rise to “second-order” moral dilemmas (i.e., even though he maintains that moral dilemmas proper are distinct from MD):

[T]he nurse in moral distress actually faces dilemmas. The dilemmas are not about patient autonomy in conflict with medical benefit; instead, the dilemmas are about what to do when one knows the right thing to do and faces institutional obstacles and the conflicting judgments of others. Thus, moral distress immediately gives rise to moral dilemmas of a different kind; perhaps, we should call these ‘second-order’ moral dilemmas. (1993, p. 544)

Here again, then, Jameton preserves the separation between MD and “proper” moral dilemmas. There is nothing inherently objectionable about distinguishing between initial and reactive distress or first- and second-order moral dilemmas, but because these distinctions presuppose Jameton’s narrow construal of MD as rooted in remediable institutional constraints, they further entrench a view of MD as a fixable problem rather than as a reflection of our condition as finite moral agents.

## Broader views

Jameton’s 1984 account of MD is still hugely influential, and broader definitions are generally advanced with revisionary tenor. The first of two significant broadenings emphasizes *internal* constraints, or those arising from within an HCP rather than the external, institutional context in which they work (see Table 1). For example, Wilkinson (1989) observes that “constraints also come from within, e.g., need for approval, self-doubt, having been taught to follow orders, and feelings of powerlessness” (1989, p. 515).^10^ Building on Wilkinson’s insights, Pendry (2007) defines MD as “the physical or emotional suffering that is experienced when constraints (internal or external) prevent one from following the course of action that one believes is right” (p. 217).

We take this attention to internal constraints to be a step in the right direction, but we would go further, since the constraints that characterize the examples of moral uncertainty and moral dilemmas from the previous section are not straightforwardly internal or institutional. That said, some theorists who emphasize internal constraints may interpret the category more broadly than as agential flaws or shortcomings. Woods (2014), for instance, illustrates internal constraints by noting that “it is often difficult to know what the right thing to do is because there are always a number of conflicting interpretations and possibilities” (p. 127). As we have argued, difficulties in determining the right course of action may arise for two reasons: (a) the HCP has certain agential shortcomings (e.g., lacking moral competence), or (b) there is genuinely no single right course of action (e.g., a moral dilemma or unavoidable moral uncertainty). It is unclear whether Woods would classify the latter situations in terms of internal constraints. Pendry (2007) treats internal constraints as arising from HCPs’ “belief systems,” and the relevant situations do arise from HCPs’ belief systems, broadly understood: one would not have the relevant moral deontic experiences without certain beliefs or moral commitments. Yet because struggling to determine the right course of action in these situations does not indicate any agential flaw, the relevant constraints differ from the internal constraints that are typically emphasized; they are better understood as features of the human condition–testaments to the principled limits of our agency–than as internal constraints.

The characterizations of MD that we have surveyed so far are unified in tying MD to institutional and/or internal constraints. The second significant broadening of Jameton’s characterization relaxes this relationship and acknowledges other causes of MD (see Table 1). Hanna (2004) proposes understanding MD as an “umbrella category,” and she locates the distressing experiences associated with moral dilemmas and moral uncertainty under this umbrella (p. 4).^11^ Similarly, Fourie (2015) denies–on grounds similar to our own–that internal or external constraints are necessary for MD:

If we continue using constraint as a necessary condition of moral distress, there is a risk of neglecting cases of distress arising from morally relevant causes other than constraint. At the least, if we are interested in health care practitioners’ morally relevant experiences of distress, we need to acknowledge there are forms of distress which cannot be linked to constraint but which may be caused by moral challenges such as conflict or uncertainty. (p. 96)

She therefore defines MD as “a psychological response to morally challenging situations such as those of moral constraint or moral conflict, or both” (Fourie 2015, p. 97).

We welcome this reconsideration of the relationship between MD, moral uncertainty, and moral dilemmas, but any attention to MD as a distinct experience with a particular structure drops out of view; MD becomes a catch-all for distress associated broadly with moral difficulty. Morley et al. (2019) acknowledge a similar concern, noting that MD has been expanded to the point that the term “lacks conceptual clarity” and refers “unhelpfully to a wide range of phenomena and causes” (p. 647). However, their positive proposal–that MD be defined as “the combination of (1) the experience of a moral event, (2) the experience of ‘psychological distress’ and (3) a direct causal relation between (1) and (2)” (2019, p. 646)–does little to remedy this problem, since it too neglects the moral phenomenology of MD. In other words, both Fourie (2015) and Morley et al. (2019) construe MD in the reductive psychological terms we criticized earlier–as distress *caused by* a moral event or situation, rather than as a distinctly moral form of distress–with the result that they lose sight of the moral experience identified by Jameton and preserved in constraint-based accounts.

In a follow-up study, Morley et al. (2020) attempt to flesh out their definition of MD by using “feminist interpretive phenomenology” to “explore and analyse the experiences of critical care nurses” (p. 1297). This approach appears promising. However, their inquiry remains constrained by their prior definition, which directs attention primarily to the moral events that give rise to MD. Accordingly, their central finding is that “psychological distress can occur in response to a variety of moral events. The moral events identified as causing psychological distress in the participants’ narratives were moral tension, moral uncertainty, moral constraint, moral conflict and moral dilemmas” (Morley et al. 2020, p. 1297). This is informative, but it is an account of the *causes* of critical care nurses’ experiences rather than an account of their structure *as experiences*. Indeed, by characterizing the relevant phenomenon in advance as “psychological distress,” the authors effectively foreclose the possibility of uncovering the distinct moral phenomenology of MD.

Finally, Campbell et al. (2016) consider six cases that they argue should be recognized as MD but that fall outside the scope of constraint-based accounts. Accordingly, they propose an understanding of MD that is meant to encompass all six cases:

*Moral distress* = _df_ one or more negative self-directed emotions or attitudes that arise in response to one’s perceived involvement in a situation that one perceives to be morally undesirable. (p. 6)

Campbell et al. take the primary objection to this definition to be practical–that it might, in virtue of its breadth, impede efforts “to identify and remedy [MD] in real-life contexts” (2016, p. 7). In response, they grant that “it might prove difficult to conduct research on moral distress in general when it is so broadly conceived,” but “it seems perfectly possible for researchers to overcome this problem by specifying a particular type of moral distress and making that their object of study” (p. 7). We think this response assuages the practical objection, but the more pressing objection is that their definition is less a characterization of MD than a retreat from the attempt to characterize it as a distinct moral experience–one specific enough to elicit a charged recognition in those who have experienced it.

For instance, one of Campbell et al.’s examples is a case of *bad moral luck*, or a situation in which an agent’s actions are beyond reproach but nevertheless cause or contribute to a bad outcome:

A psychiatrist pushes hard to get his patient to take a medication that he believes will help to address her intractable depression. The patient is initially reluctant, but he eventually persuades her. Two weeks later, she takes an intentional overdose of the medication, which results in her death. The psychiatrist feels terrible about his role in the patient’s suicide and wonders whether he did the right thing. However, after reviewing the case, he continues to think that he did exactly what someone in his position should have done, given the evidence available to him at the time. Even so, he feels great distress about the consequences of his action. (2016, p. 5).

We think this is an excellent example because it challenges the thought that MD always results from a flaw or shortcoming. However, we would aim to tease apart the different layers of the psychiatrist’s experience, as constraint-based views may shed light on one of these layers, in which case it would make sense to identify that layer as MD.

Consider two such layers. First, the psychiatrist might feel morally responsible for the patient’s death. This sense of responsibility, which can survive the recognition that one was not at fault, is generally referred to as “agent-regret” (Williams 1981, pp. 27 − 8).^12^ In Williams’ classic example, a lorry driver hits and kills a child through no fault of his own (1981, pp. 27 − 8). Williams observes that the driver will likely feel an anguished sense of responsibility for the child’s death–not, strictly speaking, guilt or self-blame, but something that goes well beyond the standard regret spectators are likely to feel.

Second, however, the psychiatrist might have a different experience before he sorts out the senses in which he is and is not responsible: he may experience a moral requirement to prevent the patient from overdosing even though he cannot heed this requirement because the patient has already overdosed. He is constrained, as it were, by his inability to go back in time.^13^ Depending on how this experience manifests, we might describe the psychiatrist as in a state of shock; what overwhelms him, though, is the utter impossibility of doing what he experiences as categorically required.

If one takes seriously prior efforts to articulate MD as a distinct moral experience, it seems preferable to identify this latter experience as MD than to maintain that both experiences are versions of MD simply because they involve “negative self-direct emotions or attitudes… in response to one’s perceived involvement in a situation that one perceives to be morally undesirable” (Campbell et al. 2016, p. 6). Doing this would require a modification we have already mentioned–namely, embracing a broader understanding of constraints–but this is a more measured response than abandoning the effort to characterize MD as a distinct moral experience.

## Morally necessitated but blocked agency

We have argued that accounts tying MD exclusively to institutional and/or internal constraints are too narrow, while accounts that expand MD to encompass any distress or otherwise negative emotions arising from morally challenging circumstances lose sight of an important phenomenon. In response, we propose that MD is best understood as a distinct moral experience with a specific structure: it is the emotionally charged experience of morally necessitated but blocked agency. More colloquially, it is the felt collision of a moral “*I must*” with an “*I can’t*.” This proposal preserves what constraint-based accounts rightly emphasize–the felt obstruction of moral agency–while allowing that the source of the obstruction can be something other than an institutional or agential problem that might be remedied. Thus, it avoids reducing MD to psychological distress caused by morally challenging circumstances, since what is essential to MD is not merely the cause of one’s distress but the experience of morally necessitated but blocked agency. Moreover, it situates MD alongside moral uncertainty and moral dilemmas as phenomena that we must, at some level, accept rather than resolve, and so as something that speaks to our moral vulnerability as human beings.

According to this proposal, MD is structured by two experiential elements: (1) a felt moral requirement and (2) the concurrently experienced impossibility of fulfilling that requirement. Earlier, we described this first element as a moral deontic experience, or an experience of a particular response as morally required (or, conversely, of a particular response as morally forbidden). Importantly, though, this experience need not be epistemically secure: the HCP may be mistaken about what is required, and they may later revise their initial judgement. We have not made any claims about when or which moral deontic experiences reflect moral deontic realities. As long as the requirement presents itself to the agent with the force of moral necessity, though, it plays the relevant experiential role (in much the same way that as long as something presents itself to an agent as threatening, it plays the relevant experiential role in characterizing their fear).

Second, the agent experiences themself as obstructed from heeding this requirement–for instance, because of institutional hierarchies or legal constraints, because the agent cannot muster the courage or tolerate the risk of losing their job, or because the situation is a moral dilemma or the outcome irreversible. What unifies these examples, then, is not their source but their ability to intercept an experience of requirement. Thus, the experience of a constraint need not be epistemically secure either; the question is not whether the agent truly lacks the ability to heed the requirement, but whether they experience themself as lacking this ability. In some cases, reflection may reveal that the obstruction was not quite the barrier that one initially thought; in others, it may prove tragically real. For the purposes of characterizing MD, however, what matters is the experienced obstruction of agency.

Finally, these two elements must be experienced simultaneously. An HCP might first calmly register a moral requirement and later register that they cannot fulfill it, but MD is only experienced when these elements overlap. Because it is this simultaneity that generates the distinct experience of MD, moreover, it is not merely psychological distress but the anguished or panicked sense that one is constrained from doing what one takes to be morally required.

This proposal also allows us to reframe a central dispute in the healthcare literature on MD and potentially reconcile the disputants: the disagreement is not fundamentally about whether constraints matter, nor about whether moral dilemmas and uncertainty belong within the scope of MD. Rather, it concerns what counts as a constraint and how. Jameton and those who follow him correctly emphasize that institutions can constrain HCPs from responding in ways they take to be morally required. Likewise, theorists who emphasize internal constraints are right that agential features can be experienced as constraints as well. And finally, those who include cases of moral uncertainty, moral dilemmas, and bad moral luck correctly observe that the laws of nature and our condition as finite beings can present themselves to us as constraints rather than as considerations that nullify our experiences of requirement.

What has been missing, then, is attention to the moral phenomenology of MD and so an understanding of constraints that is informed by that phenomenology (as opposed to, e.g., the need for institutional reform). Moreover, attending to this phenomenology reveals a third kind of constraint: *human constraints*, or constraints that arise simply from our condition as finite beings. We navigate the world with limited knowledge and power, we are culturally and temporally situated and subject to the laws of nature more generally, and we have limited control over what we care about and how, such that we could not–even if we wanted to–pull our normative experiences into perfect alignment with these limitations.

The prevalence of human constraints is perhaps best–if painfully–captured by the fact that less than 50% of adverse events in healthcare are possible to predict or prevent (De Vries et al. 2008; Rafter et al. 2015). Medical and technological improvements might shift this proportion, but they will never render every adverse event predictable or preventable; we simply cannot predict the future in the way that this would require.

However, we would also like to put forward a more subtle–and perhaps more contentious–kind of human constraint. All of the deontic moral experiences that we have discussed so far have been experiences of an *action* as either required or forbidden. However, deontic moral experiences can also pertain to our *feelings*, as we sometimes experience our emotions as morally impermissible.^14^ Consider, for instance, situations in which a patient’s agency has been diminished as a result of a disease or disorder or the side effects of treatment, such as loss of cognitive capacity as in Alzheimer’s disease; decreased motivation and indecisiveness as in Major Depressive Disorder; extreme fatigue caused by chemotherapy; and loss of functions of the most intimate kind that can result from treatment for prostate cancer in men and breast cancer in women (Safarinejad et al., 2013; Eziefula et al. 2013; Boquiren et al. 2015; and Chambers et al. 2017). Imagine, moreover, that an HCP’s emotional response to a patient in any of these situations–say, feelings of disgust or superiority or even simply pity–“fits” the patient’s diminished state, and yet distresses the HCP because it is disrespectful of the patient, who, after all, should be viewed as dignified no matter what non-ideal form of agency they retain (Solbakk 2021). The HCP may experience a moral requirement to revise their emotions yet be prevented, by their deeply-engrained emotional sensibilities, from heeding this requirement. They might therefore experience MD for merely feeling these emotions (i.e., even if they do not outwardly express them), in much the same way that people are sometimes distressed by the relief they feel after the slow death of a loved one. But unless one is in the business of trying to overhaul ordinary human emotional sensibilities, there is nothing to do but sit with this kind of distress.

As this example highlights, attending to the moral phenomenology of MD clarifies what is at stake. If MD were always the product of institutional or agential flaws, then remediating these flaws would be the appropriate goal. But because certain instances of MD arise from the finitude of our moral agency itself, the goal must involve acceptance as well. Recognizing this does not undermine efforts at reform but situates them within a broader acknowledgment of our moral vulnerability as agents whose experiences of moral necessity and possibility do not always align.

## Conclusion

Since Jameton introduced “moral distress,” the concept has traveled far and wide. Each move captures something important. Yet without accountability to the moral structure of the experience under consideration, these moves either mistake a particular cause of MD for its essence or stretch MD so widely that it loses its distinctiveness.

We have suggested that MD is comprised of a moral deontic experience and a constraint that prevents one from heeding that experience, and that MD is therefore best understood as the anguished or panicked sense that one is constrained from doing what one takes to be morally required. This suggestion preserves the central insight of constraint-based views as well as the insight of those who have been motivated to reach beyond institutional and internal constraints. By recognizing human constraints, moreover, we can reconcile these strands without reducing MD to generic psychological distress that arises in a moral context.

Insofar as HCPs are morally responsive agents, they will sometimes find themselves pinned between a moral “*I must*” and an “*I can’t*.” MD names this experience. It therefore reveals our moral vulnerability to institutions and to our own moral composition, as well as the moral vulnerability inherent in moral agency for creatures like us. In Williams’ (1981) words, it reminds us that “One’s history as an agent is a web in which anything that is the product of the will is surrounded and held up and partly formed by things that are not” (p. 29).

## Footnotes

1 In a later publication, Jameton acknowledges earlier uses of the term (2013, p. 298). However, since his 1984 reintroduction of the term initiated the current explosion of interest in MD, it will be our starting point.

2 Two previous reviews that trace understandings of MD since Jameton introduced the term are McCarthy and Deady (2008) and McCarthy and Gastmans (2015).

3 Our key words included ‘moral distress’, ‘moral residue’, ‘moral failure/error’, ‘moral injury’, ‘culpable’, ‘culpability’, and ‘fallibility.’ Thank you to university librarian Marie Susanna Isachsen at UiO for assistance with this search.

4 We repeated our initial search on multiple dates, with the final search occurring on June 4, 2024. In total, our searches identified 2482 publications. 545 publications were removed either because they did not have abstracts (485 publications) or because they were published before Jameton’s 1984 work (60 publications). The remaining abstracts (1937 abstracts) were screened to determine whether the publication proposed a new or refined definition of MD. 58 publications were retained for review on these grounds.

5 As we discuss later, some of those who take a psychological approach to MD characterize it in terms of a range or bundle of psychological states rather than a single psychological state. However, we will generally adopt singular formulations for ease of exposition.

6 The claim that MD is a distinct moral experience is more modest than the claim that it constitutes a distinct psychological kind. The former requires only that, from a first-person perspective, we can recognize multiple experiential episodes as sharing a common core despite differences in detail–where what counts as a “detail” will depend on various contingencies, including our values and associative histories. The latter, by contrast, would require adopting a substantive account of what makes something a psychological kind–for example, a shared functional role, as in Putnam (1967), or a distinct homeostatic property cluster, as in Boyd (1991)–and then arguing that MD satisfies those criteria.

7 See Street (2008), whose account of “normative experiences” we return to later.

8 We include “or an otherwise *correct* moral take” in order to leave space for moral sentimentalism–the view that the emotions play an essential role in the anatomy of morality–and so acknowledge the possibility that “knowing the right thing to do” does not boil down to having true moral beliefs but rather fitting moral emotions. We remain neutral on this question in the current paper.

9 Going forward, we will omit the negative case when it would be clunky to include.

10 Hamric et al. (2012) add lack of assertiveness and lack of understanding of the full situation to this list (p. 2).

11 See also McCarthy and Deady (2008), who label MD as a “cluster concept or umbrella concept” (p. 259).

12 Enck and Condley (2023) also discuss agent-regret in the healthcare context and argue that it is distinct from MD.

13 See Tessman (2017, pp. 116-8) for further discussion of this kind of case.

14 Interestingly, Jameton was among the first to gesture towards these experiences in the healthcare context, but only very briefly: “What is the appropriate response to the angry, demanding, or unappreciative patient? Should nurses ever express feelings of hostility toward patients? These conflicts raise questions of self-assertion and of the extent to which personal roles can be used to realize personal objectives” (1977, p. 22).
